# Ruminant Brucellosis in the Kafr El Sheikh Governorate of the Nile Delta, Egypt: Prevalence of a Neglected Zoonosis

**DOI:** 10.1371/journal.pntd.0000944

**Published:** 2011-01-11

**Authors:** Yamen M. Hegazy, Amgad Moawad, Salama Osman, Anne Ridler, Javier Guitian

**Affiliations:** 1 Department of Veterinary Clinical Sciences, Royal Veterinary College, London, United Kingdom; 2 Department of Animal Medicine, Faculty of Veterinary Medicine, Kafr El Sheikh, Egypt; 3 Department of Microbiology, Faculty of Veterinary Medicine, Kafr El Sheikh, Egypt; Swiss Tropical Institute, Switzerland

## Abstract

**Background:**

Brucellosis is a neglected tropical zoonosis allegedly reemerging in Middle Eastern countries. Infected ruminants are the primary source of human infection; consequently, estimates of the frequency of ruminant brucellosis are useful elements for building effective control strategies. Unfortunately, these estimates are lacking in most Middle East countries including Egypt. Our objectives are to estimate the frequency of ruminant brucellosis and to describe its spatial distribution in Kafr El Sheikh Governorate, Nile Delta, Egypt.

**Methodology/Principal Findings:**

We conducted a cross-sectional study in which 791 sheep, 383 goats, 188 cattle milk tanks and 173 buffalo milk tanks were randomly selected in 40 villages and tested for the presence of antibodies against *Brucella* spp. The seroprevalence among different species was estimated and visualized using choropleth maps. A spatial scanning method was used to identify areas with significantly higher proportions of seropositive flocks and milk tanks. We estimated that 12.2% of sheep and 11.3% of goats in the study area were seropositive against *Brucella* spp. and that 12.2% and 12% of cattle and buffalo milk tanks had antibodies against *Brucella* spp. The southern part of the governorate had the highest seroprevalence with significant spatial clustering of seropositive flocks in the proximity of its capital and around the main animal markets.

**Conclusions/ Significance:**

Our study revealed that brucellosis is endemic at high levels in all ruminant species in the study area and questions the efficacy of the control measures in place. The high intensity of infection transmission among ruminants combined with high livestock and human density and widespread marketing of unpasteurized milk and dairy products may explain why Egypt has one of the highest rates of human brucellosis worldwide. An effective integrated human-animal brucellosis control strategy is urgently needed. If resources are not sufficient for nationwide implementation, high-risk areas could be prioritized.

## Author Summary

Brucellosis is a zoonosis of mammals caused by bacteria of the genus *Brucella*. It is responsible for a vast global burden imposed on human health through disability and on animal productivity. In humans brucellosis causes a range of flu-like symptoms and chronic debilitating illness. In livestock brucellosis causes economic losses as a result of abortion, infertility and decreased milk production. The main routes for human infection are consumption of contaminated dairy products and contact with infected ruminants. The control of brucellosis in humans depends on its control in ruminants, for which accurate estimates of the frequency of infection are very useful, especially in areas with no previous frequency estimates. We studied the seroprevalence of brucellosis and its geographic distribution among domestic ruminants in one governorate of the Nile Delta region, Egypt. In the study area, the seroprevalence of ruminant brucellosis is very high and has probably increased considerably since the early 1990s. The disease is widespread but more concentrated around major animal markets. These findings question the efficacy of the control strategy in place and highlight the high infection risk for the animal and human populations of the area and the urgent need for an improved control strategy.

## Introduction

Brucellosis is one of the most common zoonotic diseases worldwide, and as such poses a major threat to human health and animal production [1]–[2]. It is considered a neglected zoonosis by the World Health Organization (WHO), and has been identified as having the highest public health burden across all sections of the community; livestock keepers, consumers of livestock products and general population [3].

Several Middle Eastern and central Asian countries have recently reported an increase in the incidence of human brucellosis and the appearance of new foci [4]. Among the Middle East countries, Syria, Saudi Arabia, Iraq, Iran and Turkey have reported the highest annual incidence rates of human brucellosis worldwide with the exception of Central and Inner Asian countries; 160, 21, 28, 24 and 26 cases/100,000 persons-years at risk, respectively [4].

In Egypt, brucellosis is endemic among humans and domestic ruminants [5], and it has recently been found that catfish in the Nile Delta region can be naturally infected with *Brucella melitensis* [6]. There is a lack of information on the frequency of human brucellosis at the national level in Egypt, with few available figures obtained mainly from small scale surveys and hospital-based studies [4]. In the Nile delta region, the incidence was estimated at 18 cases/100,000 population in 2000 [7] and the seroprevalence within a village in the Gharbia governorate was estimated at 1.7% in 2003 [8]. To try to address the lack of reliable information, Jennings et al. [9] used population-based surveillance data to estimate the frequency of human brucellosis in one of the Upper Egypt governorates (Al Fayoum). They reported an incidence of 64 and 70 cases /100,000 population in 2002 and 2003 respectively, and found that hospital based surveillance identified less than 6% of human brucellosis cases.

Reliable estimates of the frequency of brucellosis among ruminants in Egypt are also lacking despite an official control policy based on annual serological testing of all ruminant species over 6 months of age. Failure to test all eligible animals every year as per official guidelines, and non-random selection of herds/flocks or animals to be tested, are the reasons why accurate estimates of the seroprevalence of ruminant brucellosis in the country are not available [10]. The largest survey conducted so far across all governorates was carried out from 1994 through 1997, when 40% of the total ruminant population in the country was serologically tested against *Brucella* spp. as part of a national brucellosis surveillance and control project funded by United States Agency for International Development (USAID). The seroprevalence of brucellosis was estimated then at 0.9%, 0.3%, 1.8% and 8.2% of the cattle, buffalo, sheep and goat population, respectively [5], [11]. A recent study of 126 herds found 17.2%, 26.6% and 18.9% of the cattle farms, sheep flocks and goat flocks tested to be seropositive [12], but no information is given about the selection of herds/flocks which seem to have been conveniently or purposively selected.

Ruminant species infected with *Brucella* spp. are known to be the primary source of human infection in Egypt and other endemic countries [5], [13]. In Egypt, the close contact between farmers and their animals due to the predominance of small scale farms, occupational exposure of farmers, veterinarians and butchers to infected animals and consumption of unpasteurized milk and dairy products are considered to be the major risk factors for human infection with *Brucella* spp. [8], [9], [14]. This suggests that measures aimed at reducing the occurrence of brucellosis in animals are the most effective means of reducing human infection [15]. In order to undertake any control program, good quality data regarding the seroprevalence of infection among animals is highly desirable. As previous experiences in different countries have demonstrated, the more appropriate combination of specific measures for the control of ruminant brucellosis depends on the baseline frequency of infection; this is reflected in guidelines issued by international organizations such as the Food and Agriculture Organization (FAO) and the World Organization for Animal Health (OIE) [16]. The objectives of the present study are therefore to estimate the seroprevalence of ruminant brucellosis and to describe its geographic distribution in one of the largest governorates of the Nile Delta region, the Kafr El Sheikh governorate.

## Materials and Methods

A cross-sectional study was carried out between January and July 2008 to estimate the seroprevalence of brucellosis first, among dairy cattle and buffalos and second, among sheep and goats reared in Kafr El Sheikh governorate; an area of high density of livestock in the Nile Delta. The governorate consists of 10 districts and 206 villages. This study was approved by the Ethics and Welfare Committee of The Royal Veterinary College, London, UK.

### Target population and sampling strategy

Up to 85% of the cows and buffaloes in Egypt are reared as household animals in small herds typically of less than five animals. They have frequent contact with sheep and goats, which are sometimes also kept as household animals in the farmers' houses [17]. A typical village in the study area would have several milk tanks (usually between five and 15 for cow's milk and the same number for buffalo's milk), one milk collector is usually responsible to manage one to three tanks for each species, to which farmers take twice a day the milk surplus that they want to sell. Milk collectors have three main channels to sell the milk they collect. First, they can sell milk directly to local consumers in the same village. Second, they can sell milk to food shops in nearby villages which sell it to consumers as fresh unpasteurized milk. Third, there are several small and a few large dairy processing plants which buy milk from collectors and either sell it as fresh milk, cream or butter without heat treatment or as pasteurized milk and milk products [18]. Not all farmers sell all their milk surplus to milk collectors, some sell milk and dairy products directly in the local markets and this milk is typically sold without heat treatment.

The majority of small ruminant flocks in the villages were kept as small sheep flocks, goat flocks, or mixed flocks of both species managed by sheepherders [17], [19]–[20]. One sheepherder would often keep sheep from a number of different owners; as a result animals from different households are part of the same flock for grazing and breeding during most of the year.

A multistage random sampling strategy was used to select cattle milk tanks and individual sheep and goats within the governorate. The first level sampling units in this study were the villages, the second level sampling units were the cattle milk tanks and the individual sheep/goat.

The sampling frame consisted of the 206 villages within the governorate. In each district (stratum), the number of villages to be sampled was proportional to the size (total number of villages) of the district (sampling proportional to size). Within each selected village, sample frames of milk collectors and of sheep/goat flocks managed by individual sheepherders were constructed with the help of the village veterinarians and some farmers.

Milk collectors were selected using simple random sampling and for each of them a milk sample for each species was taken from the milk tank. If the collector managed more than one tank for either species, one tank for each species was selected by the investigator by pointing at one of the tanks without applying any defined rule (haphazard selection). All the sheep and goats reared in the village were considered as belonging to a single flock: the “village flock”. However, the management of this “village flock” is typically the responsibility of a small number of sheepherders, among which the village flock is divided for purpose of management. The number of sheep and goats to be sampled within one village was equally divided between the existing sheepherders and individual animals were selected when passing through an opening with a flock-size specific sampling interval, or, when this was not possible, the investigator pointed at individual animals for sampling without a specific rule.

### Laboratory techniques

One liter of whole milk was collected from each selected bulk milk tank and kept at room temperature for three to six hours until transported to the laboratory. Fifteen ml of milk was placed in a sealed McCartney bottle and preserved at −20°C until tested. Whole blood samples were collected from all selected individual sheep and goats using centrifuge tubes and transported directly to the laboratory where the sera were separated after centrifugation and preserved at −20°C until tested.

Milk samples were tested using an indirect enzyme linked immunosorbent essay (iELISA) for the presence of *Brucella* spp. antibodies. Serum samples were tested using Rose Bengal Plate test (RBPT). Only serum samples that were seropositive by RBPT were sent to the Animal Health Research Institute (AHRI) in Cairo for confirmation using Complement Fixation test (CFT). Serum samples which gave positive results in both tests were considered seropositive, while negative samples were those which gave negative results to either RBPT or CFT. All serological kits and reagents used were obtained from the OIE Reference Centre and an FAO/WHO Collaborating Centre for Brucellosis at the Veterinary Laboratories Agency, Weybridge, United Kingdom. All techniques were done according to the instructions of the manufacturer.

### Diagnostic test performance

A range of likely values of sensitivity (Se) and specificity (Sp) of the RBPT and CFT tests when applied at the individual animal level and of the iELISA test when applied to bulk milk samples were obtained from the literature: RBPT (0.72≤Se≤1; 0.8≤Sp≤1); CFT (0.81≤Se≤1; 0.8≤Sp≤1); iELISA (0.95≤Se≤1; 0.92≤Sp≤1) [21]–[25].

For purpose of sample size calculation fixed values of Se and Sp were used for each test: For the series combination of RBPT and CFT we used Se = 0.9 and Sp = 0.9 and for the iELISA we used Se = 0.95 and Sp = 0.92.

For purpose of seroprevalence estimation the likely values of combined sensitivity (CSe) and specificity (CSp) of the series interpretation of RBPT and CFT were calculated as CSe (0.78) and CSp (0.99), respectively in another study by the authors (Y. Hegazy, unpublished. data). In this study, most likely values of CSe and CSp were obtained using simulation. The values reported in the literature for the Se and Sp of individual tests and mentioned above were used as input probability distributions in the simulation.

For estimation of the true seroprevalence of milk tanks we used values sensitivity (Se_ELISA_) = 0.98 and specificity (Sp_ELISA_) = 0.98.

### Sample size

The number of milk tanks to be sampled was calculated in order to estimate the proportion of seropositive tanks with 95% confidence and 6% absolute error (*d*), for an expected proportion of seropositive tanks of 50%. The necessary sample size (*N*) was calculated as in [26] as following:
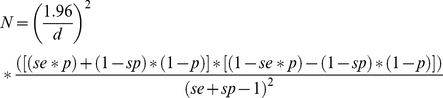



The resulted number of samples needed was multiplied by a design effect to consider the multistage level clustering of the sampling design. The design effect was calculated as:
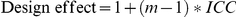



Where *m* is the number of animals per cluster and *ICC* is the intracluster (intravillage) correlation coefficient. In the absence of suitable estimates of ICC for brucellosis under local husbandry systems, we used ICC = 0.1, calculated from what we believed was a plausible scenario for the within and between village distribution of positive tanks.

We calculated that 35 villages in total and 5 milk tanks for each species per village would be sufficient to estimate the prevalence of seropositive tanks in the governorate with the desired absolute error. We decided to study 40 villages.

The number of sheep and goats to be sampled was calculated in order to estimate the proportion of seropositive individual animals against *Brucella* spp. with 95% confidence and 6% absolute error, for an expected seroprevalence amongst sheep and goats of 15%. The same equations as for the calculation of the number of milk tanks were used. Using plausible scenarios of within and between sheep and goat seroprevalence, we calculated ICC values of 0.1 for sheep and 0.05 for goats. The low value for goats reflects our expectation that due to the relatively low density of goats the impact of the presence of a positive goat within a cluster (village) would be smaller than for sheep.

We calculated that if 40 villages were to be sampled, 20 sheep and 10 goats from each village flock would be sufficient to estimate the seroprevalence among small ruminants with the desired absolute error.

### Geographic data collection

Latitude and longitude of each milk tank and small ruminant flock sampled were obtained using a Global Positioning System (GPS). An electronic map of Egypt was provided by the General Organization of Veterinary Services (GOVS) in Egypt and the locations of the main markets in the study area identified.

### Data analysis

#### Seroprevalence estimation

The apparent seroprevalence of brucellosis among individual sheep and goats (AP_s_ and AP_g_), as well as for cows and buffaloes milk tanks (AP_c_ and AP_b_), were obtained as the total number of seropositive animals or tanks divided by the total number of animals or tanks sampled. The true overall seroprevalence of brucellosis among sheep (TP_s_) and goats (TP_g_) was calculated after adjusting for the combined sensitivity (CSe) and specificity (CSp) of the serological tests as *TP* = (*AP+CSp*−1) / (*CSe+CSp*−1). The overall true seroprevalence among milk tanks of cattle (TP_c_) and buffaloes (TP_b_) was calculated in the same way by adjusting for the performance of the iELISA. Confidence intervals (CI) for TP_s_, TP_g_, TP_c_ and TP_b_ were estimated after accounting for clustering using the following equations [27]:
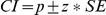



Where *p* is the seroprevalence and SE is the standard error calculated for 2 stage cluster sampling as:
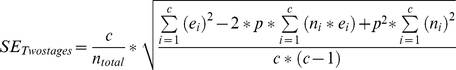



Where *c* is the number of clusters in the sample, *n*_total_ is the number of animals/tanks in the sample, *n_i_* is the number of sampled animals/tanks per cluster *i* and *e_i_* is the number of positive animal/tank per cluster *i*

In addition to overall estimates for the whole governorate, seroprevalence estimates were also obtained for each of the 40 studied villages. For small ruminants, the village flock true seroprevalence (VFTP) was calculated as *VFTP* = (*VFAP+CSp*−1)/(*CSe+CSp*−1). Upper and lower 95% confidence limits were calculated using the Wald method [28] as:
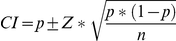
VFTP estimates and 95% confidence limits were obtained and graphically presented.

The proportion of seropositive milk tanks per village (VTTP) was calculated analogously, using Se and Sp values for the iELISA.

The proportions of seropositive villages, which has at least one seropositive sheep, goat, or milk tank, were calculated, accounting for the sensitivity and specificity of the serological tests at the village level for sheep/goats (VFCSe and VFCSp) and milk tanks (VTSe_ELISA_ and VTSp_ELISA_), as detailed below. CI for the true proportion of seropositive villages throughout the governorate was calculated using the Wald method.

#### Estimation of most likely values of sensitivity and specificity at village level

The probabilities of i) correctly identifying a village with at least one true seropositive sheep/goat (VFCSe) or milk tank (VTSe_ELISA_) ii) correctly identifying a village with no true seropositive sheep/goats (VFCSp) or milk tanks (VTSp_ELISA_) were obtained using simulation methods in @Risk version 3.5d, (Palisade Corporation, Newfield, NY, USA). The following parameters were used in the simulation: the probability of village selection was a fixed value of 0.194 (40/206); 20 sheep, 10 goats, 5 cattle milk tanks and 5 buffalo milk tanks were sampled in each village; the values of the series interpretation of CSe and CSp for RBPT and CFT (for sheep and goat samples) were used as triangular distributions with parameters 0.64, 0.78 and 0.92 for CSe and 0.97, 0.99 and 1 for CSp; the values of Se and Sp for the iELISA for milk tank samples were used as uniform distributions ranging from 0.95 to 1 and from 0.93 to 1, respectively; The probability of *Brucella* spp. seropositivity amongst individual sheep, goats and cattle milk tanks was assumed to be uniformly distributed from 0.1 to 0.15, based on the results obtained for TP_s_, TP_g_ and TP_t_. The simulations were run for 10,000 iterations, and the resultant numbers of infected villages with seropositive animals/tanks, non infected villages with seropositive animals/tanks, infected villages without seropositive animals/tanks and non infected villages without seropositive animals/tanks were used to calculate the VFCSe, VFCSp, VTSe_ELISA_ and VTSp_ELISA_.

#### Estimation of intra-village correlation

Calculation of intra-village correlation coefficients for seropositive status of individual sheep and for seropositive status of individual goats was obtained using the equation of Jung et al. [29].
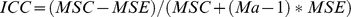


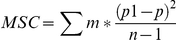


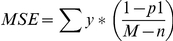


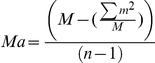


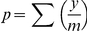



Where *MSC* is the mean square between villages (clusters), *MSE* is the mean square within villages, *n* is the number of villages, *m* is the number of either sheep or goat per village, *y* is the number of seropositive sheep, goats per village, *M* is the sum m total number of sheep, goats in all clusters, *p_1_* is the proportion of seropositive sheep, goats or per village and *p* is the overall proportion of seropositive sheep, goats or among all villages.

#### Spatial distribution of seropositive small ruminants and cattle milk tanks

District-level true prevalence estimates, obtained in the same way as for village level, were used to create choropleth maps of the geographic distribution of seropositivity in small ruminants and milk tanks within the districts of Kafr El Sheikh governorate using Arc GIS 9.2 (ESRI 2006).

A spatial scanning method was used to identify areas with significantly higher proportions of seropositive small ruminant flocks and of seropositive milk tanks (clusters). These analyses were carried out using a Bernoulli model in SaTScan v8.1.1 (www.satscan.org). Variable circular scan windows of size up to 50% of the population at risk (flocks or tanks) were used. In this analysis (global cluster test), each point location of a small ruminant flock in the study area is automatically selected as a centroid of a potential cluster. Significant clusters were identified at a *P*<0.05 by running the model for 999 simulations. Using the same settings, a focused cluster test was used to detect the presence of clusters of seropositive sheep flocks/goat flocks and seropositive milk tanks around the 9 major animal markets in the study area; in this analysis only the point locations of the markets are used as centroids of the windows.

## Results

### Seroprevalence estimation

**Figure 1:**
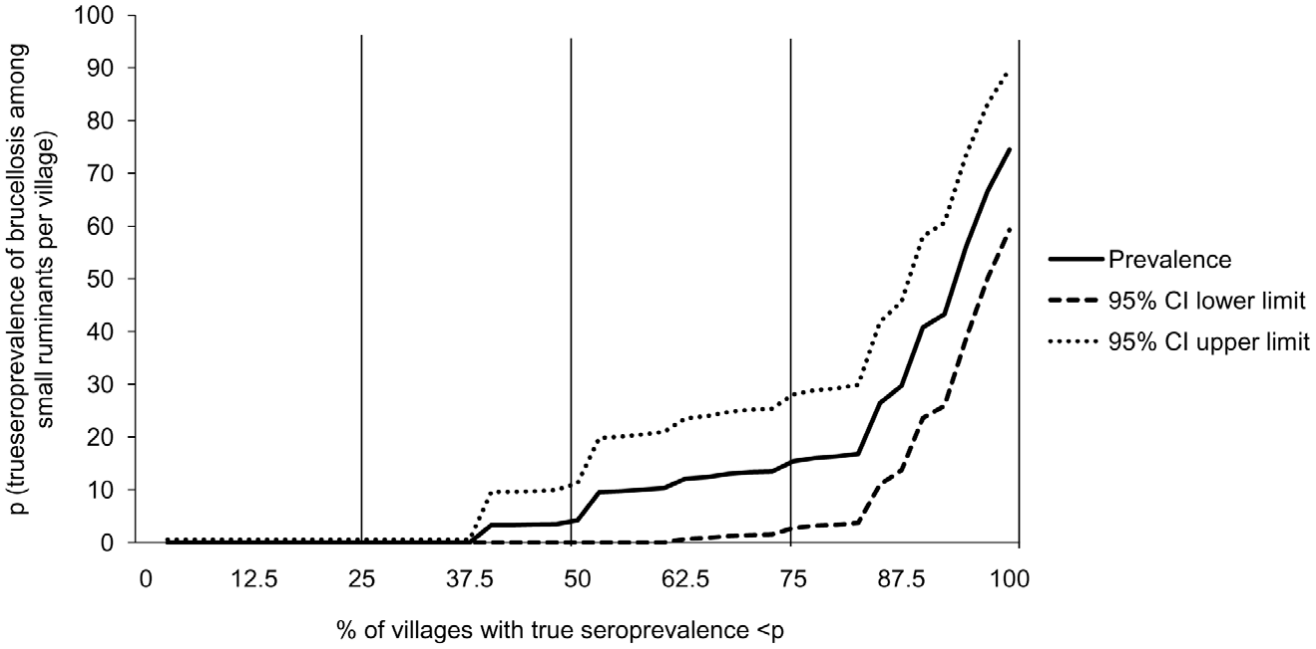
Distribution of brucellosis among ruminants in different villages of Kafr El Sheikh governorate. Distribution of within village small ruminant true brucellosis seroprevalence (VFTP) in Kafr El Sheikh governorate in a study on ruminant brucellosis in the Nile Delta, Egypt (2008).

**Figure 2:**
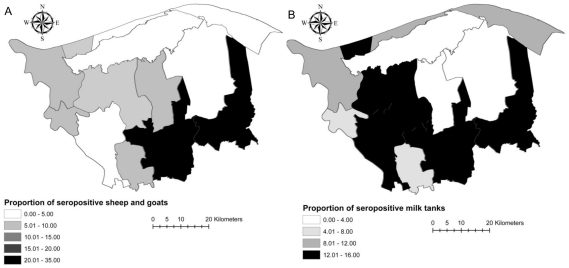
Spatial distribution of brucellosis seroprevalence among small ruminants and cattle of Kafr El Sheikh governorate. (A) Within district individual sheep and goat true brucellosis seroprevalence and (B) the true proportions of milk tanks with seropositive samples within district.

Results of serological testing of serum samples of small ruminants and milk tank samples of cattle and buffalo against *Brucella* spp. are shown in Table 1. A total of 82 (10.4%) sheep and 37 (9.7%) goats were classified as seropositive against *Brucella* spp with true seroprevalence among sheep and goats calculated as 12.2% and 11.3% respectively. The VFCSe and VFCSp were estimated at 0.93 and 0.76 for sheep and as 0.87 and 0.89 for goats, respectively. The true seroprevalence of villages with at least one seropositive sheep or goat was estimated at 41.3% and 32.2% respectively (Table 1). The true seroprevalence of villages with at least one seropositive small ruminant animal – either sheep or goat- was 60.5% (95% CI: 45.4%, 75.7%). The distribution of VFTP is shown in figure 1. The distribution of true sheep+goat brucellosis seroprevalence estimates by district is shown in figure 2.A.

**Table 1:** Results of serological testing of ruminants against *Brucella* spp. in Kafr El Sheikh governorate, Egypt.

Samples		No. tested	No. seropositve (AP)	TP (95% CI)	No infected villages* (%)	True prevalence of infected villages* (95% CI)
Serum	Sheep	791	82 (10.4%)	12.2% (8.4–16.0)	21 (52.5%)	41.3% (26.1–56.7)
	Goats	383	37 (9.7%)	11.3% (7.8–14.8)	15 (37.5%)	32.2% (17.8–46.7)
Milk tanks	Cattle	188	22 (11.7%)	12.2% (7.0–13.3)	10 (25%)	15.1% (4.0–26.2)
	Buffaloes	173	20 (11.6%)	12.0% (7.1–13.0)	10 (25%)	15.1% (4.0–26.2)

Results of testing of small ruminant serum samples and cattle and buffalo milk tank samples for the presence of antibodies against *Brucella* spp. in Kafr El Sheikh governorate, Nile Delta, Egypt (2008).

AP: Apparent seroprevalence.

TP: True seroprevalence.

*Villages with at least one seropositive sheep or goat or milk tank.

A total of 188 cattle milk tanks and 173 buffalo milk tanks were sampled in the 40 villages. Of them, 22 (11.7%) cattle milk tanks and 20 (11.6%) buffalo milk tanks were classified as seropositive against *Brucella* spp and the true seroprevalences were calculated as 12.2% and 12.0%, among cattle and buffalo milk tanks, respectively (Table 1). The VTSe_ELISA_ and VTSp_ELISA_ were calculated as 0.98 and 0.88 respectively. The true seroprevalence of villages where at least one seropositive tank was found was 38.4% (95% CI: 19.6%, 49.1%). The true seroprevalence of villages with at least one seropositive cattle milk tank was 15.1%, and the same value was obtained for the true seroprevalence of villages with at least one positive buffalo milk tank (Table 1). When considering cattle and buffalo milk tanks together, we estimated that 22 (55%) of villages had no seropositive tanks and 18 (45%) had at least one seropositive tank. Of those villages with seropositive milk tanks, 11 (27.5%) had less than 25% seropositive tanks, four (10%) of the villages had between 25% and 50% of tanks seropositive and in three (7.5%) of the villages more than half of the tanks were seropositive against *Brucella* spp. The distribution of the true proportion of seropositive milk tanks against brucellosis by district is shown in figure 2.B.

### Intravillage correlation of seropositive status against *Brucella* spp

Intracluster correlation coefficients for sheep and goat flocks were estimated at 0.21 and 0.38 respectively.

### Results of spatial analysis

**Figure 3:**
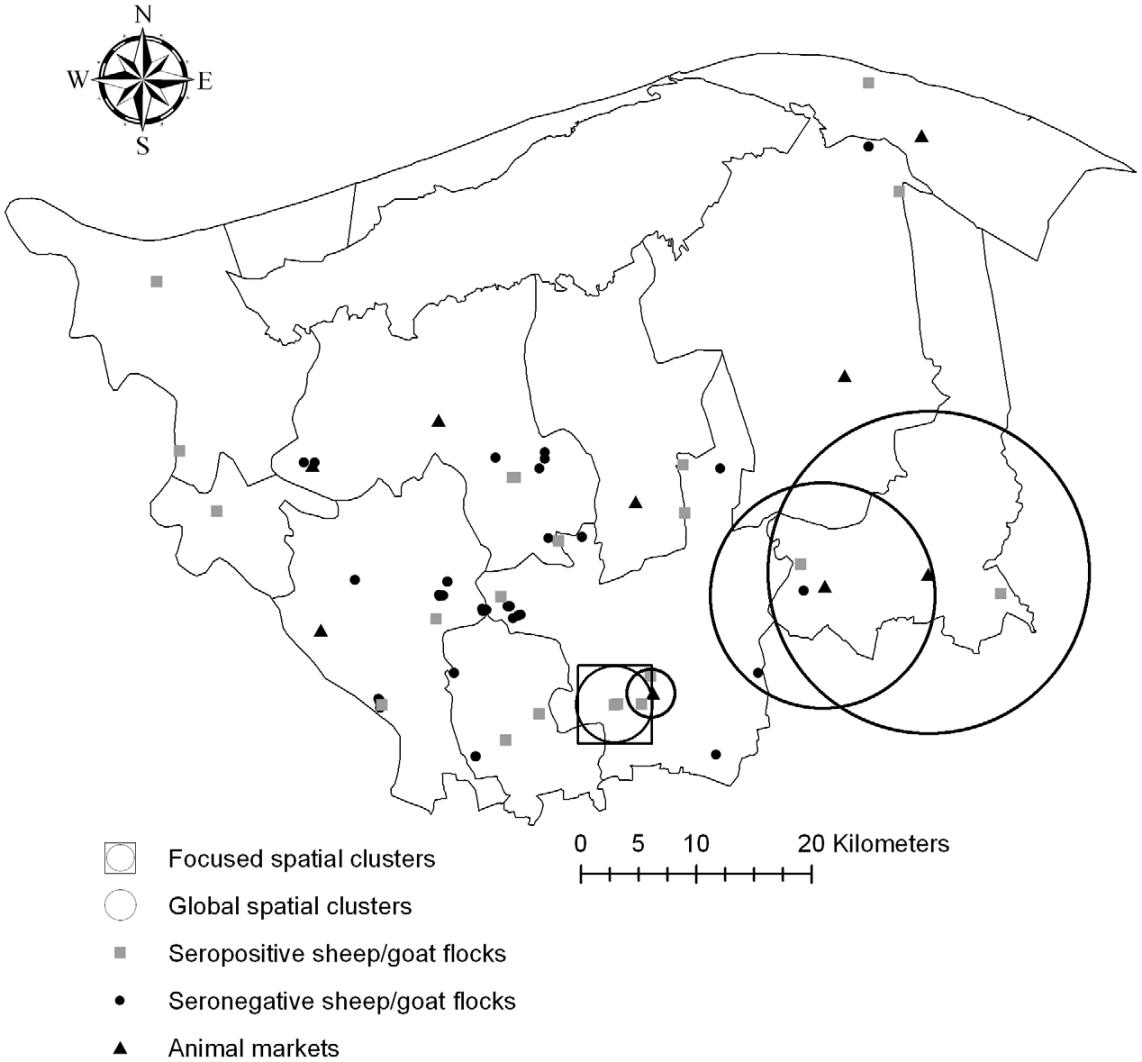
Location of clusters of brucellosis seropositive small ruminant flocks in Kafr El Sheikh governorate. The global spatial clusters of herds of sheep and goats with seropositive results against *Brucella* spp. in Kafr El Sheikh and the focused clusters of sheep and goat flocks with seropositive animals around the main animal markets. One dot or square may represent more than one flock in the map.

The southern districts of the governorate, near its capital, had the highest seroprevalence of small ruminant brucellosis (figure 2.A). Significant clustering of seropositive small ruminant flocks was identified within a 3.3 km radius area in the proximity of the capital of the governorate (*P*<0.001; figure 3). Flocks within this cluster were 3.4 times more likely to be seropositive than flocks outside the cluster. When focused scanning was conducted around major animal markets, there was also evidence of clustering of seropositive flocks around three animal markets, one near the capital of the governorate (radius 2 km, relative risk 3.4, *P*<0.001) and two in the neighboring district of Byala (radius 17 km, relative risk 3 and radius 13 km, relative risk 3; *P*<0.001) (figure 3).

Although the seroprevalence of seropositive tanks appeared to be higher in southern districts (Figure 2.B), we did not find any significant clustering of seropositive tanks across the study area.

## Discussion

The Nile Delta region has one of the highest human and ruminant densities in the world; with more than 125 person per km^2^ and more than 196 ruminant/km^2^ [30]–[31]. Most households in the region raise small numbers of cattle, buffaloes, sheep or goats which are kept in close contact with household members [14]. These animals are a source of meat and dairy products that are consumed within the same household or sold in local markets or to middlemen [14]. In the study area milk is mostly sold unpasteurized, either directly by the producers or indirectly by milk collectors or food shops. Cream and butter made by the farmers or by local dairy processing plants are also often sold without heat treatment.

The potential for human exposure to zoonotic pathogens such as *Brucella* spp. is amplified by these demographics, husbandry practices and dairy production and marketing systems, which closely tie the incidence of brucellosis in the livestock and human populations [13].

To our knowledge, this is the first formal survey with probabilistic sampling carried out with the objective of estimating the seroprevalence of ruminant brucellosis in one governorate of Egypt. The results show that brucellosis is widely spread in the study area where seroprevalence values are very high among all ruminant species, suggesting a very intense transmission within the livestock population. In fact, considering all the sheep in one village as a single flock – which, given the production system, seems appropriate – the proportion of seropositive flocks in the area (60.5%) is among the highest reported in the scientific literature for a small ruminant population [32]–[33]. Our estimates in the ruminant population are in accord with reports that identify Egypt as having one of the highest rates of human infection worldwide [9]. The coexistence with a heavily infected domestic ruminant population managed under husbandry systems such as those in place in Egypt and widespread marketing of unpasteurized milk and dairy products inevitably results in a high level of exposure of the human population.

In ruminants, *Brucella* spp. is transmitted either in-utero or by direct contact between infected and susceptible animals, therefore, a high seroprevalence is necessarily indicative of a high frequency of contacts between infected and susceptible animals. It is likely that, in the study area, a high density of ruminants with free movement of small ruminant flocks results in frequent contact between animals from different households and villages. In the absence of vaccination and other sanitary measures, this contact structure creates the necessary conditions for sustaining *Brucella* spp. infection at higher seroprevalence levels than in other regions [5], [12], [33].

Our estimates for the intravillage correlation, especially among goats, are higher than those reported in Mexico and Ireland [34]–[35]. This may suggest a high within-villages transmission of brucellosis in the study area. These estimates could be used for study designs in future surveys to insure a proper sample size and better prevalence estimates.

Although our study does not differentiate between *Brucella* strains, in Egypt, the main isolate in different animal species and humans is *Brucella melitensis* [5]. Given the high seroprevalence in small ruminants, it is likely that cattle act as spill-over hosts of *Brucella melitensis*. [36]. The recent isolation of *Brucella melitensis* from Nile Catfish in different regions of the Nile Delta points out the potential extent of *Brucella melitensis* infection pressure currently in the area [6].

Comparisons of our estimates with the results of the 1994–1997 national control campaign have to be made with great caution, since that nationwide study was not designed to generate unbiased prevalence estimates for the governorates. However, if the 1994–1997 estimates did not heavily underestimate the existing seroprevalence of infection at the time (an assumption that seems reasonable to us), the seroprevalence of ruminant brucellosis in the study area has increased considerably in the last 10 years. The establishment of infection as endemic at such high levels across the different species is also indicative of the ineffectiveness of the control program that has been in place since 1981. Recent reports have shown the inability of the test and slaughter element of the program to test more than 7% of the total ruminant population each year in this governorate as well as the noncompliance with the official vaccination and quarantine policies [9]–[10]. The need for a better implement the existing official strategy or the consideration of other control measures that are better suited to the high frequency of infection across all species, the available resources and the structure of the production systems are highlighted by our results [10], [16], [37].

Across this study, taking into account the imperfect performance of the serological tests, the calculated true village flock prevalence was lower than the apparent prevalence and vice versa for the animal prevalence. In addition, we estimated the positive and negative predictive values at the flock level at 72% and 94.2% respectively (data not shown). Therefore, ignoring the imperfect performance of the serological tests would result in an overestimation of the proportion of infected flocks and an underestimation of the proportion of infected animals. Control programs for brucellosis that are based on the apparent prevalence estimates will result in considering many non infected villages as false positives.

In the light of the local dairy processing and marketing practices outlined above, the finding of 38.4% of milk tanks seropositive against *Brucella* spp., suggests that unpasteurized milk and dairy products may be a major source of exposure of the general population to *Brucella* spp, including people not keeping livestock in their households. These findings should be considered by public health authorities in the study area and highlight the need for coordinated action between public health and veterinary services. Interventions that would effectively reduce the prevalence of ruminant brucellosis in the Nile Delta would benefit not just livestock keepers but the general population. Therefore, a combined strategy for the control of brucellosis designed and implemented in collaboration by veterinary and public health authorities would be justified and could result in a better allocation of resources [38].

Finally, this study shows that the distribution of brucellosis among different ruminant species within the Kafr El Sheikh governorate is spatially heterogeneous, with clustering of the infection around the capital of the governorate and the main animal markets. The finding of higher seroprevalence towards the south of the study area may be associated to higher livestock density compared to the northern part of the governorate (more dependent on fishing) and to the proximity to the largest animal market in the Nile Delta region in the Gharbia governorate. The spatial clustering of infection suggests that there may be potential for the prioritization of control activities in certain areas. By applying different control measures at specific locations it may be possible to maximize public health benefits and to minimize spread of the infection to areas with lower seroprevalence [39]. A recent FAO/WHO report on *Brucella melitensis* in Eurasia and the Middle East proposes zoning/compartmentalization within a country as one of the generic disease control measures that could be applicable to the control of *Brucella melitensis* [37]. Such a control strategy was one of the elements of the program successfully applied for the eradication of brucellosis in Chile [40]. For compartmentalization to be effective it has to be accompanied by a biosecurity border that could be difficult to implement in Egypt given the intensity of unregulated animal movements [5]. However, consideration should be given to this approach and others that may be more realistic than achieving elimination by testing a limited fraction of the population with slaughtering of seropositive reactors in the absence of vaccination, which is the strategy currently in place in the area [5], [10].

The results here presented are highly compatible with an intensity of infection transmission within livestock higher than in any other ruminant population studied in Egypt and nearby Middle Eastern countries. Our reference population was restricted to only one of the five governorates of the Nile Delta, mainly because of the availability of relatively detailed information concerning the implementation of brucellosis control activities in this specific governorate in previous years. However, husbandry practices are similar across the entire Nile delta region and thus the situation in neighboring governorates is not likely to differ considerably. Similar surveys in other parts of the country or a survey with nationwide coverage could be a worthwhile investment to provide the basis for the redesign and implementation of control strategies that are more appropriate to the baseline level of infection, structure of the production systems and availability of resources. The sampling strategy presented in this paper and some of our results including seroprevalence estimates by species, test performance indicators and values and intracluster correlation may prove useful in the design of such surveys. Our experience here presented suggests that even relatively small surveys based on inexpensive diagnostic strategies such as bulk tank milk testing for antibodies may provide enough evidence to justify changes in the existing control strategies.

In the light of the results here reported and other concordant published evidence, we recommend that serious consideration should be given to an integrated human-animal brucellosis control program in the Nile delta region and that surveys aimed at estimating the frequency of ruminant brucellosis are carried out in other parts of the country such as Upper Egypt and the dessert governorates.
